# Dysgerminoma Masquerading as Gestational Trophoblastic Neoplasia

**DOI:** 10.1155/2023/1901858

**Published:** 2023-02-08

**Authors:** Conner Blackwell, Shian McLeish, David Iglesias, Shannon D. Armbruster

**Affiliations:** ^1^Department of Obstetrics and Gynecology, Virginia Tech Carilion School of Medicine, Roanoke, VA, USA; ^2^Department of Obstetrics and Gynecology, Division of Gynecology Oncology, Virginia Tech Carilion School of Medicine, Roanoke, VA, USA

## Abstract

**Background:**

Persistent elevation in beta-human chorionic gonadotropin (*β*-hCG) following a pregnancy is concerning for gestational trophoblastic neoplasia (GTN). However, the differential diagnosis should remain broad during the evaluation process.

**Case:**

A 34-year-old G3P3 presented with elevated *β*-hCG four months after cesarean delivery with bilateral tubal ligation. The patient was treated with methotrexate for a presumed new ectopic pregnancy. Due to persistent *β*-hCG elevation, she received actinomycin-D for GTN treatment. After completing chemotherapy, her *β*-hCG increased. The patient underwent a laparoscopic hysterectomy with unplanned left oophorectomy due to its nodular appearance at the time of surgery. Pathology confirmed a dysgerminoma of the ovary and benign uterus.

**Conclusion:**

Although dysgerminomas are uncommon, they should be considered when *β*-hCG levels remain elevated despite therapies for more common pathologies.

## 1. Introduction

Persistent elevation of beta-human chorionic gonadotropin (*β*-hCG) following pregnancy presents a diagnostic challenge due to the broad differential diagnosis list, including several etiologies requiring close monitoring and treatment (Table 1). In the months following pregnancy, a positive urine pregnancy test can represent new intrauterine pregnancy, ectopic or molar pregnancy, gestational trophoblastic neoplasia (GTN), or etiologies unrelated pregnancy, such as *β*-hCG producing ovarian germ cell tumors, quiescent *β*-hCG, and heterophilic antibodies.

The case presented here highlights the diagnostic evaluation of a persistently elevated *β*-hCG following pregnancy, the diagnosis and management of GTN, and, finally, the treatment and prognosis of stage IA dysgerminomas. This patient's journey to ascertain the correct diagnosis of dysgerminoma can inform other gynecologists and gynecologic oncologists who may encounter a similar scenario. The patient consented to the publication of her story.

## 2. Case Presentation

A 34-year-old G3P2 underwent a scheduled repeat cesarean delivery and bilateral tubal interruption following an uncomplicated pregnancy. At the time of delivery, both adnexa had a grossly normal appearance. She had an uncomplicated postoperative course.

Four months later, she presented to the clinic following a positive home urine pregnancy test. A confirmatory serum quantitative *β*-hCG was 141 IU/L. A transvaginal ultrasound (TVUS) demonstrated a 3 cm right ovarian mass and a thin endometrium with no intrauterine contents. Intramuscular methotrexate (50 mg/m^2^) was given for a presumed ectopic pregnancy. There was an insufficient decrease in the day 7 *β*-hCG measurement (113 IU/L), so a second dose of methotrexate was given. Seven days later, her *β*-hCG remained elevated at 96 IU/L, and TVUS showed an unchanged right ovarian mass.

Approximately 1 week later, her gynecologist performed a diagnostic laparoscopy, bilateral salpingectomy, bilateral ovarian cystectomy, and uterine dilation and curettage. The final pathology confirmed a right-sided corpus luteum, left-sided follicular cyst, and benign proliferative fragments from the endometrium.

Two weeks later, the serum *β*-hCG remained elevated at 158 IU/L, prompting referral to a gynecologic oncologist. Following the initial consultation, the patient's case was reviewed with experts at a National Gestational Trophoblastic Disease Referral Center. A computerized tomography (CT)-chest/abdomen/pelvis showed no evidence of pelvic or metastatic disease. Magnetic resonance imaging (MRI) of the pelvis confirmed no lesions. Due to suspected GTN, actinomycin-D 1.25 mg/m^2^ IV was administered every 2 weeks for 4 cycles with serial assessments of serum *β*-hCG to follow response during her treatment. Before the first cycle of actinomycin-D, *β*-hCG peaked at 350 IU/L but then normalized prior to cycles 3 and 4 (*β*-hCG = 1.6 and 0.7 IU/L, respectively). Actinomycin-D was discontinued after cycle 4, and surveillance was initiated.

Weekly *β*-hCG assessments were performed, and values were noted to increase following discontinuation of treatment, peaking at 77 IU/L after 6 weeks of monitoring. Imaging was repeated, including a CT-chest/abdomen/pelvis and brain MRI, which demonstrated that an abnormal appearance of the junctional zone of the uterus near the prior Cesarean section scar was noted, but no additional abnormalities were identified. The decision was made to proceed with a hysterectomy.

**Figure 1:**
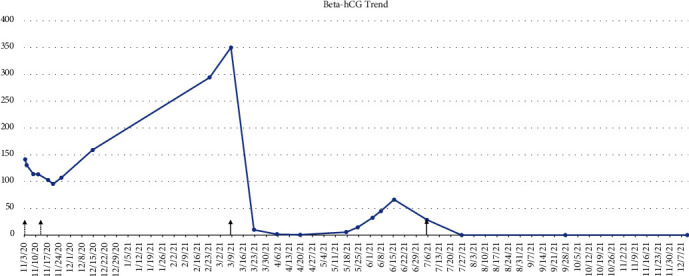
Beta-human chorionic gonadotropin (*β*-hCG) trend. The first two arrows indicate the doses of methotrexate. The third arrow indicates the initiation of 4 doses of actinomycin-D that occurred every 2 weeks. The fourth arrow indicates one additional dose of actinomycin-D given just prior to hysterectomy with left oophorectomy. *β*-hCG has remained 0 since surgery.

After one additional cycle of actinomycin-D per expert recommendation, the patient underwent a total laparoscopic hysterectomy with a left oophorectomy due to the elongated, nodular appearance of the ovary. The pathology confirmed a stage IA dysgerminoma of the ovary and benign findings in the uterus with no evidence of GTN. Three weeks later, the *β*-hCG was <0.5 IU/L and has remained undetectable for 5 months.  Figure 1 demonstrates the *β*-hCG trend during her work-up and treatment.

## 3. Discussion

The management of persistent *β*-hCG elevation requires prompt lab work and imaging. If the patient reports a positive home urine pregnancy test, this result must be confirmed with a quantitative serum *β*-hCG assessment. An analysis of common home urine pregnancy tests showed that these tests detect levels ranging from 5.5 to 22 IU/L [1]. If the patient has an elevated serum *β*-hCG, TVUS is warranted to evaluate for a new intrauterine pregnancy, ectopic pregnancy, or molar pregnancy [2]. An abnormal appearance of the adnexa may also suggest a *β*-hCG-producing ovarian mass, including ectopic pregnancy, dysgerminoma, or ovarian embryonal carcinoma. For patients with a persistent low-level elevation in serum *β*-hCG that has not yet had a urine pregnancy test, this should be performed to rule out *β*-hCG heterophilic antibodies, which do not enter the urine.

When the initial work-up does not identify the etiology of the elevated *β*-hCG, the patient should have serial *β*-hCG levels drawn. International Federation of Gynecology and Obstetrics (FIGO) 2000 guidelines established four diagnostic criteria for GTN: (1) four or more plateaued *β*-hCG concentrations over 3 weeks; (2) increasing *β*-hCG levels for three or more measurements for at least 2 weeks; (3) histologic diagnosis of choriocarcinoma; and (4) elevated *β*-hCG for 6 months or longer [3].

Once a patient meets criteria for GTN, a chest X-ray should be performed to evaluate the lungs, which are a more frequent location for metastatic GTN [3]. Low risk GTN has high rates of response to actinomycin-D [4]. Hysterectomy is not typically required for GTN, but this patient's atypical response to treatment and abnormal imaging prompted surgical removal of the anticipated source of disease.

Dysgerminomas account for only 2% of all ovarian neoplasms, but they are the most common malignant germ cell tumors. They are often found in young women with 75% identified in women between 10 and 30 years old. Dysgerminomas are associated with elevations in lactate dehydrogenase, and only 15% are associated with *β*-hCG production [5]. A review of the literature shows that most women are symptomatic with bloating or pain. Most appear as large solid masses on imaging, and approximately half present with fluid in the pouch of Douglas [6–8]. To our knowledge, there are no other published reports of a patient found to have a dysgerminoma during the surgical management of GTN, who did not previously have symptoms or imaging findings.

Dysgerminomas are most often stage I at diagnosis with 90% occurring in only one ovary [9]. Patients with stage IA disease have 5-year survival rates greater than 95% after unilateral oophorectomy. There is no survival benefit for patients undergoing omentectomy or lymphadenectomy [10]. As a result, the patient presented in this case did not require re-operation given her post-operative *β*-hCG normalization and negative imaging. This is consistent with current guidelines published by the National Comprehensive Cancer Network (NCCN) [11].

The patient presented in this case was recommended to undergo surveillance for the first year with exams and *β*-hCG check every 2–3 months and a CT of the abdomen and pelvis every 3–4 months. Screening with *β*-hCG every 6 months and annual CT imaging will continue for 5 years at intervals as recommended by the NCCN [12]. This case highlights the importance of considering alternative diagnoses when presumed low risk GTN does not respond to standard therapy. Although ovarian germ cell tumors are uncommon, it is important to consider all potential etiologies when patients with persistent elevated *β*-hCG following pregnancy.

## Figures and Tables

**Table 1:** Differential diagnosis of elevated beta-human chorionic gonadotropin (*β*-hCG).

Obstetric	New intrauterine pregnancy
	Missed abortion
	Ectopic pregnancy
	Molar pregnancy
Oncologic	Gestational trophoblastic neoplasia (placental site trophoblastic tumor, epithelioid trophoblastic tumor, choriocarcinoma, and invasive moles)
	Embryonal carcinoma
	Dysgerminoma
	Non-gynecologic cancers (kidney, bladder, lung, breast, and gastrointestinal)
Others	Heterophilic antibodies
	Peri-menopausal pituitary production
	Quiescent *β*-hCG
	Exogenous *β*-hCG

## Data Availability

Data supporting this research article are available from the corresponding author or first author on reasonable request.
